# Giant villous adenoma of the sigmoid colon: an unusual cause of homogeneous, segmental bowel wall thickening

**DOI:** 10.1259/bjrcr.20200016

**Published:** 2020-06-16

**Authors:** Jeffrey Sacks, Seymour Atlas, Alar Enno, Leonardo Santos, Jeremy Humphries, Alexander Kirwan

**Affiliations:** 1 Department of Radiology, Bankstown-Lidcombe Hospital, Sydney, NSW, Australia; 2 University of New South Wales, Sydney, NSW, Australia; 3 Department of Pathology, Liverpool Hospital, Sydney, NSW, Australia; 4 Department of Gastroenterology, Bankstown-Lidcombe Hospital, Sydney, NSW, Australia; 5 Department of Radiology, Westmead Hospital, Sydney, NSW, Australia

## Abstract

Colonic adenomas are commonly encountered lesions that are a precursor of colorectal cancer. Of these, villous adenomas are a rarer, more advanced subtype that are larger in size than tubular adenomas and have a higher risk of malignant transformation. We present a patient with a giant villous adenoma of the sigmoid colon identified on CT as homogeneous segmental bowel wall thickening.

## Clinical presentation

A 66-year-old female of Vietnamese background presented to our institution on numerous occasions over a period of 1 year with symptoms including diarrhoea, back pain, suprapubic pain, dehydration and rectal bleeding. Her physical examination was normal. She was hyponatraemic with a blood sodium level of 122 mmol l^−1^ (135–145 mmol l^−1^). Her potassium level was normal. Her haemoglobin was mildly decreased at 99 g l^−1^ (120–150 g l^−1^) and her carcinoembryonic antigen (CEA) was elevated, 5.7 mg/mL (normal <3 mg/mL). Other tumour markers were negative and inflammatory markers were not significantly elevated.

## Investigations

**Figure 1.**
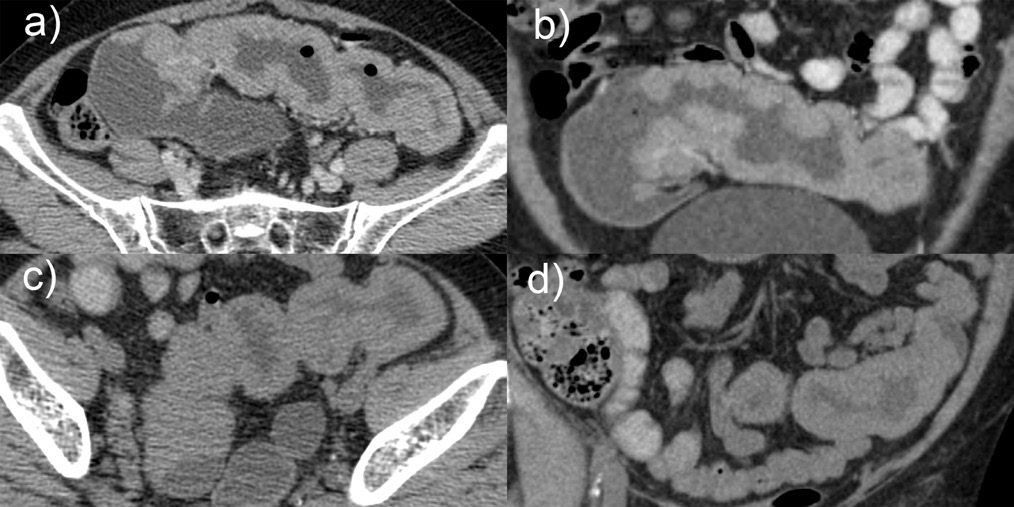
CT abdomen demonstrating segmental wall thickening of homogeneous density in the sigmoid colon. Displayed are post-i.v. and oral contrast axial (a) and coronal (b) plane images, compared with non-contrast axial (c) and coronal (d) plane images

A CT scan was performed pre-i.v. and post-i.v. contrast and with an oral contrast agent. The post-i.v. contrast scan was obtained in the portal venous phase. Circumferential bowel wall thickening, without proximal bowel obstruction, was demonstrated involving a segment of the mid-sigmoid colon measuring 150 mm in length (Figure 1). The thickened bowel wall demonstrated homogeneous density and enhancement, with a mean density of 42 HU pre-contrast and 76 HU post-contrast. There were no inflammatory changes in the perisigmoid fat, no evidence of loco-regional or distant lymphadenopathy, no ascites and no liver lesions.

**Figure 2.**
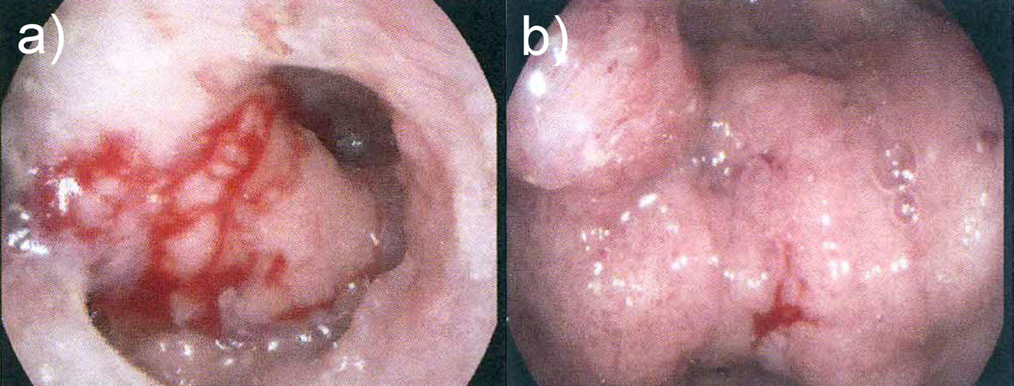
Colonoscopy images of the distal (a) and mid (b) sigmoid colon with large, frond-like, circumferential mass

Colonoscopy demonstrated a large frond-like, circumferential, villous, partially obstructing sigmoid colon mass with no active haemorrhage. It was estimated to measure 50 mm in length (Figure 2). Biopsies demonstrated fragments of a tubulo-villous adenoma with low-grade dysplasia and no evidence of malignancy.

## Differential diagnosis

Apparent thickening of the bowel wall may be considered normal, depending on the degree of luminal distension, with measurements in the colon varying from 1 to 5mm.^1,2^ Pathological thickening of the colonic wall has a broad differential and includes neoplastic, inflammatory, infectious and traumatic causes. The extent of thickening can be further described as focal (<50 mm), segmental (60–400 mm) and diffuse (>400 mm). In addition, the symmetry of thickening, contrast enhancement and the presence of pericolonic abnormalities should be evaluated..^2–4^

For patients demonstrating segmental bowel wall thickening, benign causes are more common, with ischaemia, inflammatory or infective colitis the key differentials. Colorectal adenocarcinomas more commonly present as focal thickening. Lymphoma, although less common, can present as both focal and segmental thickening..^2–4^

## Treatment

Laparoscopic high anterior resection of the rectum and distal sigmoid colon was performed, achieving macroscopic clearance. There was complete symptomatic resolution and no complications.

**Figure 3.**
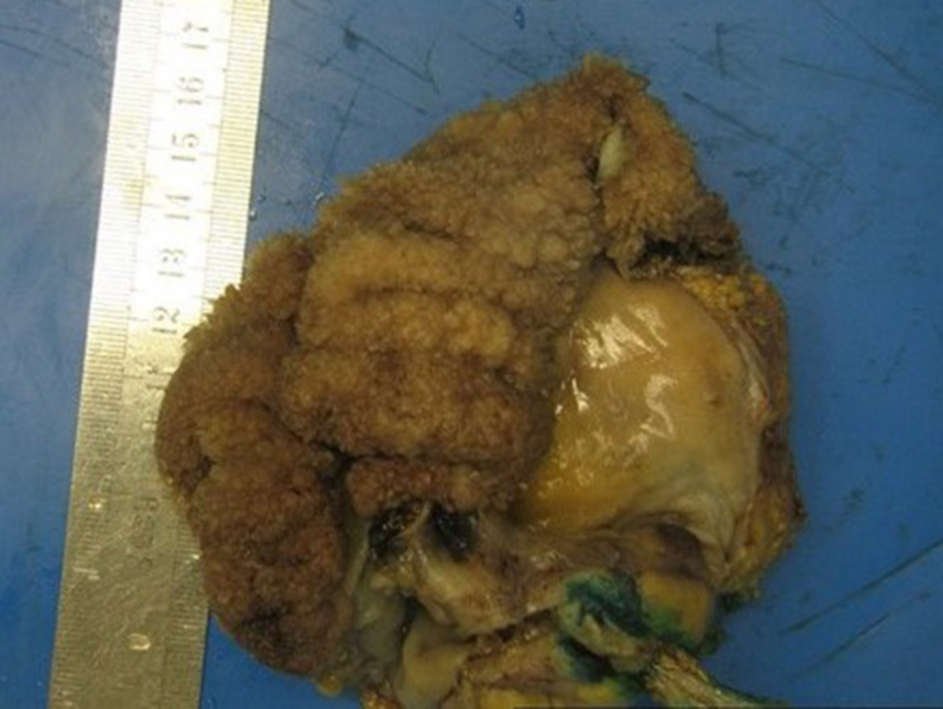
Macroscopic specimen of the rectosigmoid lesion. Note the broad *cauliflower-like* appearance

**Figure 4.**
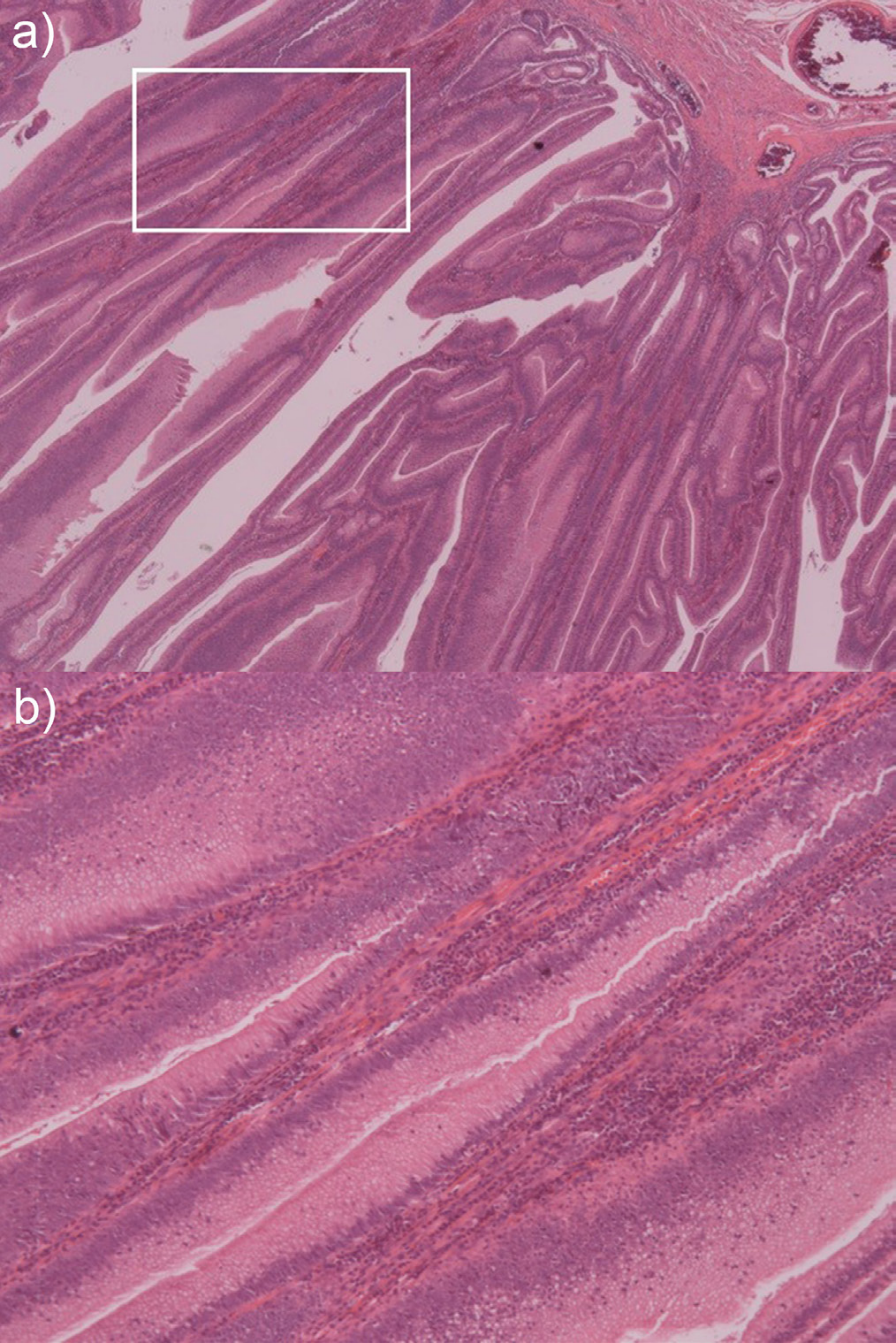
Microscopic sections of the villous adenoma. Low-power X10 view (a) and high-power view X20 (b) views demonstrating frond-like villous architecture with low-grade dysplasia

Histology demonstrated a rectosigmoid polyp with a length of 165 mm (Figure 3) and villous architecture (Figure 4) involving the entire mucosal surface, but sparing the muscularis propria. Generalised low-grade dysplasia (Figure 4) with an occasional focus of high-grade dysplasia of 2–3 mm was present. There were no areas of invasive malignancy and 19 lymph nodes were identified without evidence of malignancy.

## Discussion

Colorectal cancer is among the most common causes of cancer mortality in Western populations.^5,6^ Lesions are typically slow growing, beginning as a benign adenoma before undergoing malignant transformation over approximately 7–10 years; the *adenomacarcinoma sequence*.^1,7^ Identifying and removing the adenoma early can decrease the incidence of adenocarcinoma.^1,5,7^

Adenomas can be categorised into tubular and villous, 10% of which are villous. Villous adenomas have been described as broad, shaggy lesions with a *cauliflower-like* surface.^5^ They are frond-like and are comprised of a core of fibrovascular lamina propria lined with mucin-secreting columnar epithelium that, in larger lesions, can secrete copious quantities of mucinous fluid.^1,5,8^ Adenoma size is one of the most important markers for the risk of malignant transformation and, based on size alone, are considered advanced if they are larger than 10 mm.^7^ Villous adenomas are also larger in size than other polyps, often more than 20 mm, and can grow to greater than 100 mm. This results in an advanced subpopulation of adenomas that are at higher risk of malignant transformation.^1,5,9^ Areas of dysplasia and malignancy occur sporadically throughout these lesions, requiring surgical resection rather than biopsy.^3,10^

Although villous adenomas are usually asymptomatic, larger lesions can produce mucus secretion, diarrhoea, obstructive symptoms and, rarely, rectal bleeding.^8,11^ Villous adenomas may secrete water, sodium and potassium, felt to be secretagogue-mediated via prostaglandin E2^12^ and first described by McKittrick and Wheelock in 1954.^13^ Secretory villous adenomas that are larger, ranging in size from 70 to 180 mm, are associated with depletion syndrome due to a greater surface area for secretion.^12^ Those located distally in the colon also have less normal mucosa available for fluid resorption. This is consistent with our patient’s presentation, with evidence of diarrhoea and electrolyte depletion in the context of a large distal tumour.

Double contrast barium enema was traditionally used for radiological screening of patients with colorectal neoplasms. The findings of villous adenoma have been well described, demonstrating a sessile filling defect with a *reticular or granular* mucosal surface pattern. It has also been described as having the appearance of a *soap-bubble*. This results from wisps of barium extending between the fronds of the tumour, replacing the mucus normally present..^8,14–17^

CT has played an increasing role, however, most lesions seen on CT are detected incidentally.^1^ The literature regarding the CT appearances of villous adenoma is sparse. The first reported findings described a large contrast-enhancing mucosal-based mass with soft tissue density on its serosal aspect and near water density (3.9 HU) on its mucosal aspect.^15^ A corrugated appearance from an irregular surface coated by oral contrast was described. Subsequent descriptions include a *variegated, gyral* contrast enhancement pattern with low attenuation areas of 15–17 HU^8^ and an eccentrically located lesion with homogeneous water density occupying more than 50% of the mucosal surface of the mass.^1,9^ The low-density areas are due to the presence of mucus trapped within the lesion. Current recommendations suggest that oral contrast should be avoided, as coating the mucosal surface with barium can obscure these low density regions.^1,8,9,15^

The CT appearance of our patient’s lesion is unique when compared with previous descriptions. Although the length of greater than 150 mm is within the range described for a villous adenoma, circumferential rather than eccentric bowel wall thickening and homogeneous rather than heterogeneous and gyral enhancement are new findings. This unusual CT appearance with no similar description found in the literature should be considered additional features to those described previously and also included in the differential diagnosis of segmental bowel wall thickening in the colon.

## Learning points

1. Villous adenoma is an unusual cause for homogeneous segmental colonic wall thickening and should be included in the differential diagnosis
2. Diarrhoea and abdominal pain with bowel wall thickening and no inflammatory changes should raise the possibility of a villous adenoma.
